# Changes in Peripheral Anterior Chamber Depth of a Case of Relapsing Polychondritis with Recurrent Secondary Angle Closure Glaucoma§

**DOI:** 10.2174/1874364100802010001

**Published:** 2008-01-04

**Authors:** Kenji Kashiwagi, Toshie Furuya, Fumiko Kashiwagi

**Affiliations:** 1 Department of Ophthalmology, University of Yamanashi, Faculty of Medicine, Chuo, Yamanashi, Japan; 2 Ophthalmic Clinic Kofu, Yamanashi, Japan

**Keywords:** Relapsing polychondritis, angle closure glaucoma, auricular chondritis, peripheral anterior chamber depth.

## Abstract

A case of relapsing polychondritis showed IOP elevations three times during the follow-up due to the angle-closure mechanism. The peripheral anterior chamber depth (ACD) showed a good correlation with IOP elevation, but central ACD did not. The peripheral ACD could be more related to IOP elevation than central ACD.

## INTRODUCTION

Relapsing polychondritis is a multi-organ autoimmune disease characterized by recurrent episodes of inflammation and progressive destruction of cartilaginous tissues. Ocular manifestations including secondary open glaucoma were found in more than half of patients relapsing polychondritis [1]. We experienced a case of relapsing polychondritis with secondary ACG. This patient showed periodical elevation of intraocular pressure (IOP) due to the mechanism of angle closure. Ultra-biomicroscopy (UBM) revealed that a forward shift of ciliary body is deeply involved in this secondary IOP elevation. Here, we report a case with relapsing polychondritis whose profile of peripheral anterior chamber depth (ACD) showed significant correlation with IOP change.

## CASE

Informed consent was obtained from the patients.

**Fig. (1).**
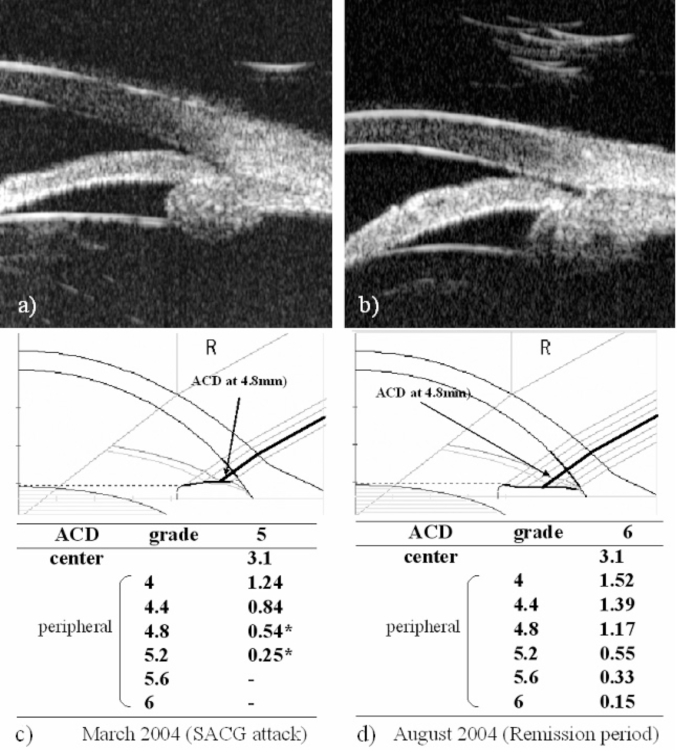
**Biometric examination of anterior ocular segment.** Ultra bio-microscopic observation shows that plateau iris configuration is observed in the both-side eyes and ciliary body is shifted forward (**a**,**b**). Results of two SPAC measurements were depicted in Fig. (**1c**,**d**). In March 2004, IOP was 34mmHg, (**c**) while in August 2004, IOP was 15mmHg without any anti-glaucoma treatment. (**d**) * : risky point for ACG automatically judged by SPAC. Bold lines indicate the ACD at the point of 4.8 mm from the central. ACD: anterior chamber depth, SACG: secondary angle closure glaucoma.

A 54-year-old Japanese female who noted blurred vision in her right eye visited a local ophthalmic clinic in 1999. She was diagnosed with IOP elevation of the right eye due to ACG and was subjected to laser iridotomy. She never showed any signs of intraocular inflammation in both eyes during this period. She was referred to Yamanashi University Hospital in October 2001 due to poor IOP control. Her corrected visual acuity was 20/20, and shallow anterior chamber but no sign of inflammation were observed in both eyes. Her right eye showed glaucomatous cupping and corresponding visual field defect. She was prescribed 0.5% timolol ophthalmic solution to be applied twice a day and 2% pilocarpine ophthalmic solution to be applied four times a day on the right eye, and one 250 mg carboxyl anhydrase inhibitor tablet to be taken orally per day. Gonioscopic observation revealed the presence of peripheral anterior synechia (PAS) occupying about 10% of the angle in the right eye and plateau iris configuration in both eyes. UBM confirmed the presence of plateau iris configuration in both eyes and a forward shift of the ciliary body in the right eye (Fig. **1a**,**b**). She underwent laser gonioplasty on both eyes. Thereafter, IOP was well controlled and no signs of intraocular inflammation were observed. In September 2002, IOP of the right eye was elevated to 44 mmHg with mild anterior chamber inflammation. Gonioscopic observation revealed angle closure and a PAS index of 20%. Blood examination revealed that some parameters associated with autoimmune diseases such as anti-nuclear antibody, serum complement (CH50), C3, anti-RNP antibody, anti-Sm antibody, and SS-A antibody, and anti SS-B antibody were elevated. She began to experience hearing impairment, tinnitus, nausea and vomiting in July 2003, and complained of acute auricular chondritis occurring at alternate times in both ears in August 2003. By considering these systemic manifestations, she was diagnosed with relapsing polychondritis.

In March 2004, the right IOP elevated at 34mmHg without any intraocular inflammatory sign. Anti-glaucoma eyedrops, topical steroid, and cycloplegic eyedrops normalized IOP. Since it was available to measure ACD using SPAC from March 2004, SPAC started to evaluate ACD periodically. As previously reported, SPAC quantitatively measures ACD in a non-contact fashion from the optical axis to the periphery leaving consecutive slit-lamp images at 0.4 mm intervals. This system is also equipped with an auto-diagnosing program for differentiating eyes with narrow angle and an auto-classifying program for categorizing eyes into twelve grades according to the ACD values [2]. Eyes with lower grade have shallower ACD. Fig. (**1c**,**d**) show SPAC results performed in March 2004 and in August 2004. SPAC evaluated the ACD at fifth grade in March 2004 and ACD measured at two peripheral points indicated the risk of ACG, while the ACD grade in August 2004 was six and all measured points were in the normal range. The central ACD of these two measurements was 3.1mm. The right eye showed IOP elevation due to Secondary ACG attack on May 20, 2006 and July 22, 2006 during the follow-up period. There were no inflammatory intraocular signs and gonioscopy revealed that the angle was closed. The peripheral ACD on these two days were very shallow. Fig. (**2**) shows the profiles of central ACD and peripheral ACD measured at the point of 4.8mm from the optic axis, and IOP during the follow up. Secondary ACG attack occurred when peripheral ACD at the point 4.8 mm from the optic axis was less than 0.6 mm and peripheral ACD change, but not central ACD change, is well complemented with IOP change.

## DISCUSSION

This patient repeatedly had bilateral relapse of acute auricular chondritis, ocular manifestations, and vestibular dysfunction with dizziness, nausea and vomiting, all of which satisfied the diagnostic criteria for relapsing polychondritis [1, 3].

More than half of the patients with relapsing polychondritis have ocular symptoms that appear in the form of recurrent episcleritis or scleritis, conjunctivitis, keratitis or uveitis [1, 4]. Additional ocular manifestations, including proptosis, periorbital edema, tarsitis and extraocular muscle palsy, retinal vasculitis, and optic neuritis, may occur [1, 4]. Although there were some reports in which relapsing chondritis sometimes induces secondary open angle glaucoma, it is rare to see a case with relapsing-polychondritis initially inducing ACG. Since UBM and other ophthalmic examinations revealed the presence of narrow angle and plateau iris configuration, we speculated that this patient initially had plateau iris configuration and slight inflammation around the ciliary body that could not be detected by routine ophthalmic examination, which pushed the ciliary body forward, resulting in angle closure. Laser gonioplasty improved the angle configuration; however, the intraocular inflammation reduced outflow facility by occlusion of the angle, which is the mechanism of the IOP elevation due to secondary ACG. It should be noted that relapsing polychondritis may be one of the reasons for secondary ACG.

The present study indicates a good correlation between peripheral ACD and IOP. As indicated in Fig. (**2**), Secondary ACG attack was induced when peripheral ACDs at 4.8mm from the center were less than 0.6 mm. Central ACD, in contrast, did not show any correlation with IOP. Therefore, peripheral ACD may be a predictor of IOP elevation.

It is well known that some intra-ocular inflammatory diseases such as Vogt-Koyanagi-Harada syndrome, sometimes result in secondary ACG and it has been postulated that the swollen ciliary body with anterior rotation and supraciliary space may induce IOP elevation [5]. Therefore, it is important to examine the angle or ciliary body to evaluate a forward shift of ciliary body using UBM. However, topical or systemic steroid administration may elevate IOP. We should be aware of possibility of steroid induced IOP elevation during the follow up period in addition to IOP change due to angle-closure mechanism.

The present study indicates that the mechanism of secondary glaucoma in case of relapsing polychondritis could be angle closure and that measurement peripheral ACD quantitatively is important to diagnose and to treat eyes with secondary ACG.

## Figures and Tables

**Fig. (2).**
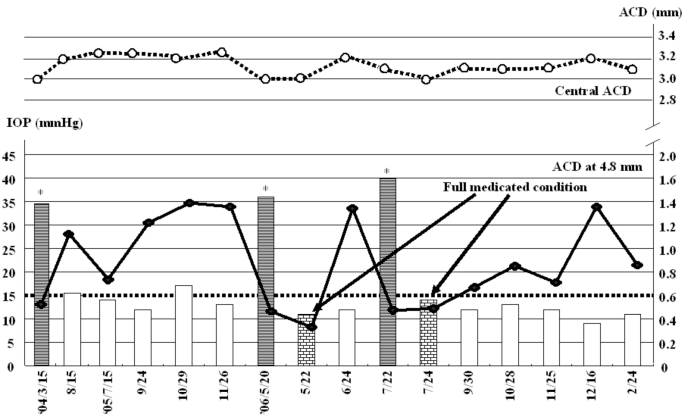
**Correlation between anterior chamber depth and intraocular pressure.** Bars indicate IOP profile during the follow up. A bold line is ACD profile at the point 4.8mm from the optic axis as indicated in Fig. (**1c**,**d**), while a dot line is ACD profile at the center. ACD: anterior chamber depth, IOP: intraocular pressure, * : secondary ACG attack.

## References

[1] McAdam LP, O'Hanlan MA, Bluestone R, Pearson CM, O'Hanlan M (1976). Relapsing polychondritis: prospective study of 23 patients and a review of the literature. The arthropathy of relapsing polychrondritis. Medicine (Baltimore).

[2] Kashiwagi K, Shinbayashi E, Tsukahara S (2006). Development of a fully automated peripheral anterior chamber depth analyzer and evaluation of its accuracy. J Glaucoma.

[3] Michet CJ Jr, McKenna CH, Luthra HS, O'Fallon WM (1986). Relapsing polychondritis. Survival and predictive role of early disease manifestations. Ann Intern Med.

[4] Isaak BL, Liesegang TJ, Michet CJ Jr (1986). Ocular and systemic findings in relapsing polychondritis. Conjunctival and episcleral injection in drug abuse. Ophthalmology.

[5] Gohdo T, Tsukahara S (1996). Ultrasound Biomicroscopy of shallow anterior chamber in Vogt-Koyanagi-Harada syndrome. Am J Ophthalmol.

